# Bilateral Idiopathic Sclerochoroidal Calcification: A Case Report

**DOI:** 10.7759/cureus.81369

**Published:** 2025-03-28

**Authors:** Boutayna Azarkan, Hamza Lazaar, Zineb Hilali, Noureddine Boutimzine, Lalla Ouafa Cherkaoui

**Affiliations:** 1 Ophtalmology A, Hopital des Specialités, CHU (Le Centre Hospitalo-Universitaire) Ibn Sina, Rabat, MAR; 2 Ophthalmology A, Hopital des Specialités, CHU (Le Centre Hospitalo-Universitaire) Ibn Sina, Rabat, MAR

**Keywords:** choroid, choroidal calcification, choroidal lesions, osteoma, retina

## Abstract

Sclerochoroidal calcification (SCC) is a rare and often asymptomatic condition characterized by the deposition of calcium pyrophosphate in the sclera and choroid. It predominantly affects elderly individuals of Caucasian descent and is most often idiopathic, although it can be secondary to systemic disorders such as hyperparathyroidism or chronic renal disease. The lesions appear as irregular, white-yellow plaques located in the mid-periphery, typically along the vascular arcades. We present the case of an 80-year-old patient with bilateral idiopathic SCC, incidentally discovered during an ophthalmologic examination. The patient’s medical history included diabetes, hypertension, and cataract surgery, and there was no history of high myopia or uveitis. Fundus examination revealed yellowish choroidal lesions bilaterally, confirmed by autofluorescence and optical coherence tomography (OCT) imaging as elevated sclerochoroidal plaques. Comprehensive systemic and laboratory evaluations excluded underlying metabolic or renal abnormalities, confirming the idiopathic nature of the calcifications. The patient remained asymptomatic, and no treatment was required. This case underscores the importance of considering SCC in the differential diagnosis of elevated choroidal lesions. It highlights the need for thorough systemic evaluations to exclude secondary causes and emphasizes the generally benign prognosis of idiopathic cases.

## Introduction

Sclerochoroidal calcification (SCC) is a rare and idiopathic pathology characterized by the deposition of calcium pyrophosphate within the sclera and choroid. It manifests as irregular, white-yellow plaques of varying sizes, located predominantly in the superotemporal mid-periphery along the vascular arcades [1]. This condition primarily affects elderly individuals of Caucasian descent, with no gender predilection [2]. Though most often idiopathic, it may, however, be secondary to disorders of hydro-electrolytic metabolism and other renal tubulopathies [3,4]. Given the nature of the diagnosis as one of exclusion, differential diagnoses, including choroidal osteomas, melanoma metastases, achromic lymphomas, Angoid streaks, and infective chorioretinitis, must first be ruled out [5].

We report a rare case of an 80-year-old patient presenting with bilateral idiopathic SCC.

## Case presentation

**Figure 1:**
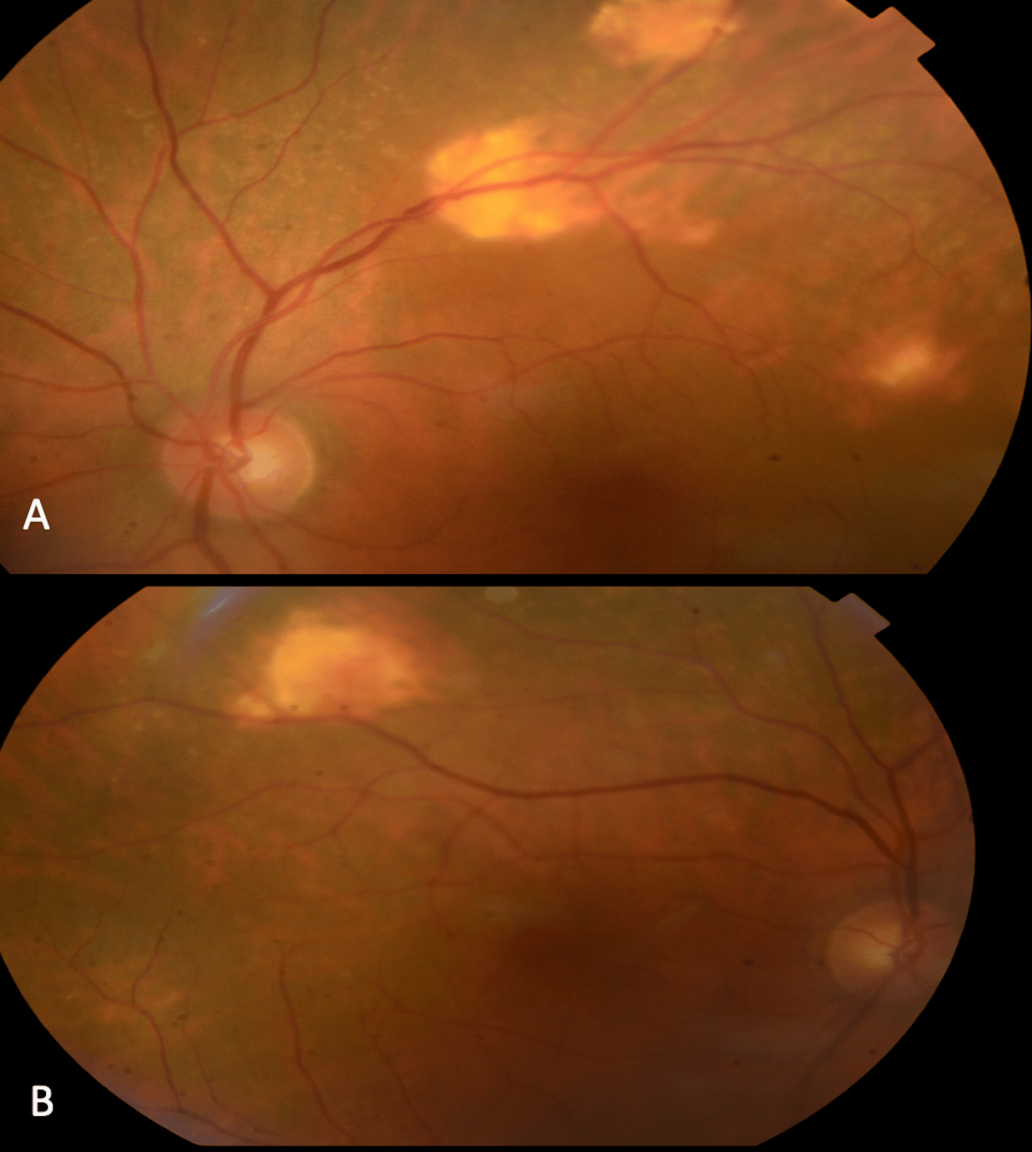
Fundus examination of the left (image A) and right eyes (image B) showing multiple sclerochoroidal calcifications

We report the case of an 80-year-old patient with a history of diabetes, hypertension, and previous cataract surgery of the left eye by phacoemulsification who presented for consultation due to complaints of floaters. The anterior segment examination revealed a best-corrected visual acuity of 4/10 on the Snellen scale in the right eye and 9/10 in the left eye. The patient has no important refractive error. An intraocular pressure (IOP) of 15 mmHg, a calm anterior chamber with good depth, normal iris and stromal appearance, and no retrocorneal precipitates were observed. The intraocular lens was clear in the left eye, and a grade II nuclear cataract was present in the right eye. Fundus examination revealed multiple yellowish choroidal lesions bilaterally (incidentally discovered) located at the superior temporal arcades with a good macular reflection, well-defined vessels, and without vascular sheathing in both eyes. They were better visualized in the left eye (Figure 1).

**Figure 2:**
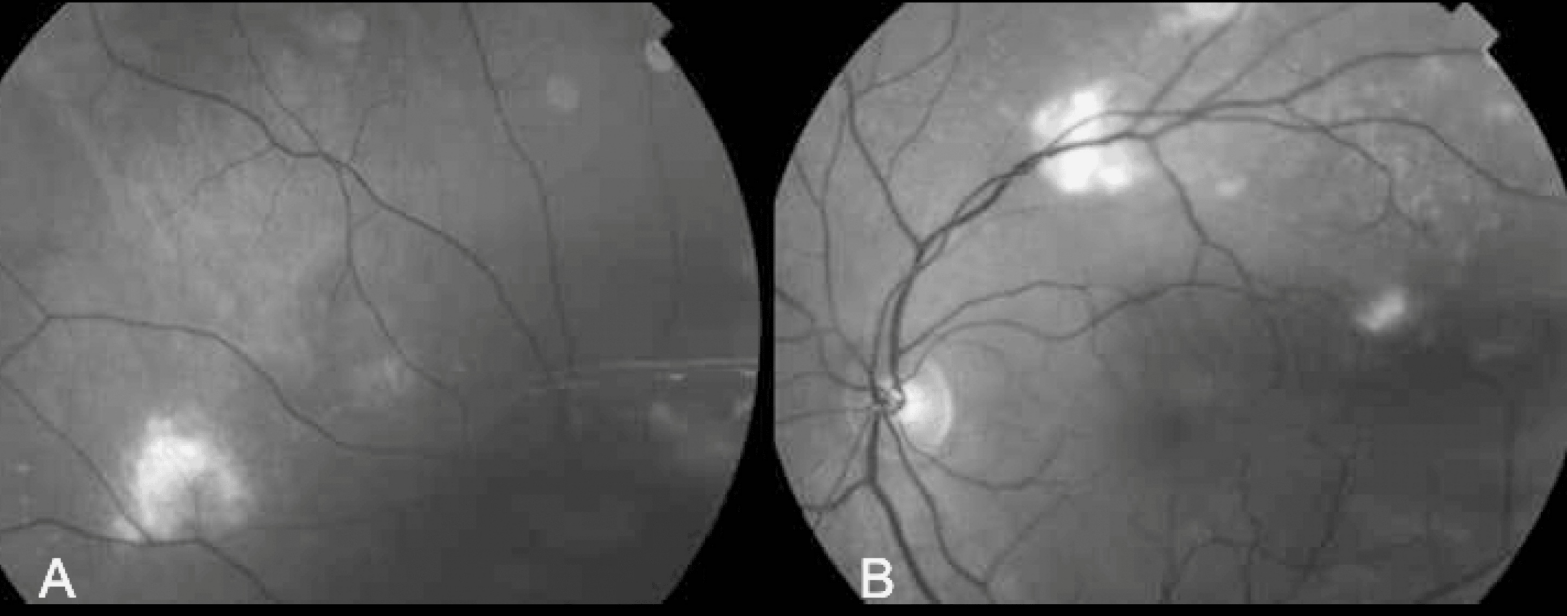
Green-light retinography of the right (image A) and left (image B) eyes showing the sclerochoroidal calcifications

The green-light fundus photograph reveals irregular whitish lesions near the vascular arcades (Figure 2).

**Figure 3:**
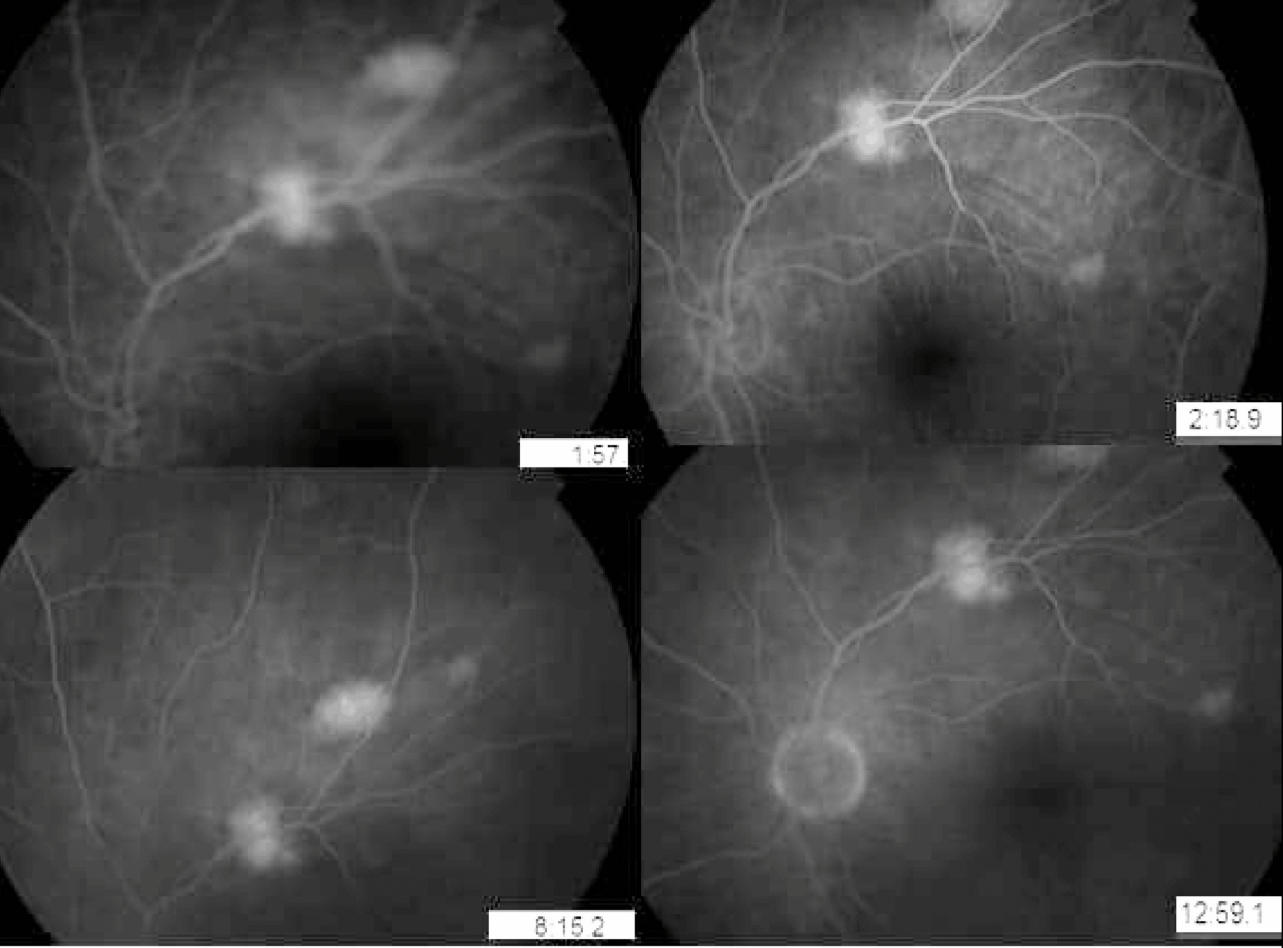
Angiography of the left eye showing hyperfluorescent lesions at early stage without diffusion

The autofluorescence image demonstrated hyperautofluorescent lesions with early-stage hyperfluorescence without diffusion in angiography (Figure 3).

**Figure 4:**
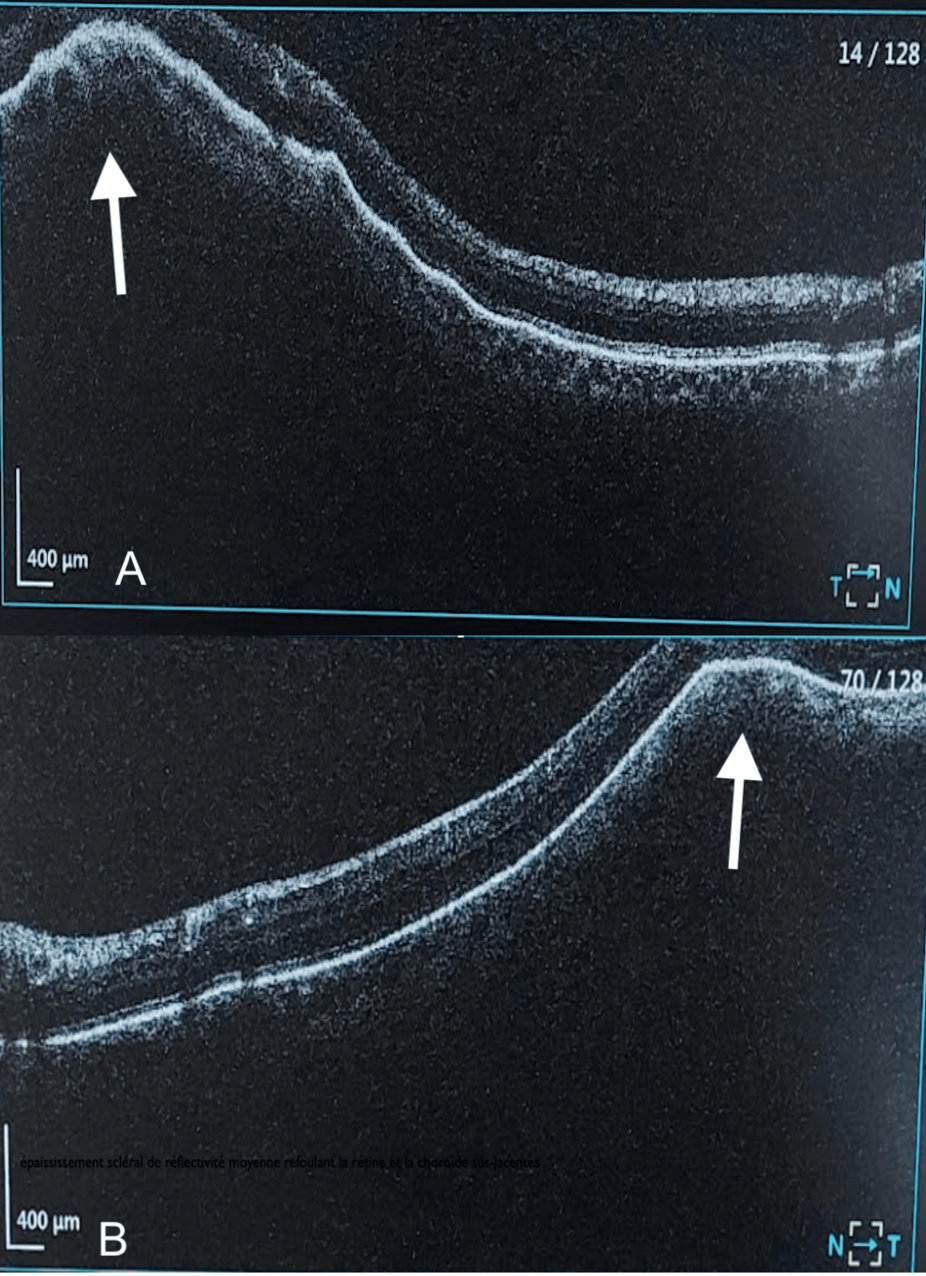
OCT centered on the right (image A) and left eyes (image B) showed a normal retina and retinal pigment epithelium, but elevated due to the sclerochoroidal lesion. OCT, optical coherence tomography.

Optical coherence tomography (OCT) centered on the lesion showed a normal retina and retinal pigment epithelium, but elevated due to the sclerochoroidal lesion (Figure 4).

A full biological workup was performed, confirming the idiopathic nature of these SCCs, in line with the majority of reported cases in the literature. Serologies for hepatitis B and C, HIV, anti-nuclear antibodies, complete blood count (CBC), and C-reactive protein (CRP) were normal; renal function tests, parathyroid hormone (PTH), T4, and serum protein electrophoresis (SPE) were within normal limits. After excluding any systemic pathology, no treatment was required. Prognosis is generally excellent.

Upon six-month follow-up, the examination and OCT imaging results remained unchanged. 

## Discussion

SCC is a rare benign intraocular lesion primarily found in elderly individuals, almost exclusively described in Caucasian patients [2]. It is idiopathic in 79% of cases and secondary in 21% [2]. It is bilateral in 52% of cases with no gender predilection [2]. The condition is most often incidentally discovered due to its typically asymptomatic nature [6]. It manifests as single or multiple irregularly bordered lesions of varying sizes, flat or slightly elevated, white-yellow in color, most commonly located in the superior temporal region, and less frequently in the superior nasal area, along the retinal vascular arcades in the mid-periphery between the equator and arcades. It can, more rarely, be found in the macular area, potentially leading to decreased visual acuity, exudation, and the formation of choroidal neovascularization [7].

Differential diagnoses primarily include choroidal melanoma, osteoma, and metastatic choroidal lesions.

Choroidal metastases are typically larger, well-defined, and often accompanied by serous retinal detachment, preferentially localized at the posterior pole [8].

Osteomas commonly appear as yellowish to orange, slightly elevated subretinal lesions with well-defined geographic margins and are most often solitary, located in the juxtapapillary or peripapillary region [9].

Choroidal melanoma is usually pigmented but can vary in pigmentation, even being amelanotic (non-pigmented). Characteristic findings include orange pigment (lipofuscin) at the retinal pigment epithelium level, exudative retinal detachment, and absence of calcium on imaging [10].

A study conducted at the Oncology Department of Wills Eye Hospital revealed that SCC lesions were often misdiagnosed as choroidal metastases (26%), choroidal melanoma (21%), choroidal nevus (11%), or an unidentified tumor (39%) [11].

SCC can be subdivided into three pathophysiological entities: idiopathic, dystrophic, and metastatic. In dystrophic calcifications, calcium crystal deposition most often occurs on necrotic or damaged tissues, or in the context of chronic inflammation, with normal phosphocalcic balance. This corresponds to band keratopathy in the cornea or optic disk drusen at the papilla [11].

In metastatic calcifications, calcium crystal deposition is secondary to a disorder in phosphocalcic metabolism seen in several pathologies. Primary hyperparathyroidism is the most common condition associated with SCC, due to hypersecretion of PTH, which leads to elevated serum calcium levels [12]. Secondary hyperparathyroidism has also been described in SCC due to chronic renal failure [12]. SCC has been reported in cases of vitamin D-related disorders, such as sarcoidosis producing 1,25-dihydroxy-cholecalciferol and vitamin D intoxication from excessive intake of vitamin D [13], and more rarely in hypovitaminosis [14].

Bartter syndrome and Gitelman syndrome, uncommon tubular metabolic alkalosis syndromes, are linked to SCCs. These autosomal recessive renal tubular diseases impair sodium and chloride transport [15].

Although often idiopathic, a thorough systemic evaluation is crucial to rule out dystrophic or metastatic calcifications due to underlying pathological conditions associated with disturbances in calcium-phosphorus metabolism [16].

A classification of SCC based on OCT imaging has been proposed by Hasanreisoglu et al. [17], categorizing SCCs into four patterns based on scleral contours: flat, rolling, rocky-rolling, and table mountain.

The management of SCC is primarily conservative, focusing on addressing any underlying conditions. In cases of choroidal neovascularization, a rare complication of SCC, anti-VEGF (vascular endothelial growth factor) injections and photodynamic therapy have shown efficacy [18].

## Conclusions

SCC should be considered as a differential diagnosis when examining patients with elevated fundus lesions. Fundoscopic clinical appearance should be compared to systemic review to determine if further workup is required. At minimum, patients with newly presenting SCC lesions should be referred for physical and complete blood work, including calcium and parathyroid function. Any systemic anomalies should be thoroughly vetted and correlated with both systemic and fundoscopic clinical findings. Consultation with endocrinology for high-risk cases is advised for the best continuity of care.
